# Molecular and morphological phylogeny of host-specific *Dactylogyrus* parasites (Monogenea) sheds new light on the puzzling Middle Eastern origin of European and African lineages

**DOI:** 10.1186/s13071-021-04863-7

**Published:** 2021-07-21

**Authors:** Michal Benovics, Farshad Nejat, Asghar Abdoli, Andrea Šimková

**Affiliations:** 1 Department of Botany and Zoology, Faculty of Science, Masaryk University, Kotlářská 2, 611 37 Brno, Czech Republic; 2 Department of Zoology, Faculty of Sciences, Comenius University in Bratislava, Mlynská dolina, Ilkovičova 6, 842 15 Bratislava, Slovakia; 3 Department of Biodiversity and Ecosystem Management, Environmental Science Research Institute, Shahid Beheshti University, Shahid Shahriari Sq. Velenjak, 1983969411 Tehran, Iran

**Keywords:** Parasites, Platyhelminthes, Phylogeography, Historical dispersion, Cyprinoidei

## Abstract

**Background:**

Freshwater fauna of the Middle East encompass elements shared with three continents—Africa, Asia, and Europe—and the Middle East is, therefore, considered a historical geographic crossroad between these three regions. Even though various dispersion scenarios have been proposed to explain the current distribution of cyprinids in the peri-Mediterranean, all of them congruently suggest an Asian origin for this group. Herein, we investigated the proposed scenarios using monogenean parasites of the genus *Dactylogyrus,* which is host-specific to cyprinoid fishes.

**Methods:**

A total of 48 *Dactylogyrus* species parasitizing cyprinids belonging to seven genera were used for molecular phylogenetic reconstruction. Taxonomically important morphological features, i.e., sclerotized elements of the attachment organ, were further evaluated to resolve ambiguous relationships between individual phylogenetic lineages. For 37 species, sequences of partial genes coding *18S* and *28S* rRNA, and the *ITS1* region were retrieved from GenBank. Ten *Dactylogyrus* species collected from Middle Eastern cyprinoids and *D. falciformis* were de novo sequenced for the aforementioned molecular markers.

**Results:**

The phylogenetic reconstruction divided all investigated *Dactylogyrus* species into four phylogenetic clades. The first one encompassed species with the “varicorhini” type of haptoral ventral bar with a putative origin linked to the historical dispersion of cyprinids via the North African coastline. The second clade included the majority of the investigated species parasitizing various phylogenetically divergent cyprinid hosts. The morphological and molecular data suggest the ancestral diversification of the species of this clade into two groups: (1) the group possessing the haptoral ventral bar of the “cornu” type, and (2) the group possessing the “wunderi” type. *Dactylogyrus* diversification apparently occurred in the Middle East, which is indicated by the presence of species with all morphotypes in the region. The last two clades included species parasitizing cyprinids with an East Asian origin, and species possessing the “magnihamatus” type of ventral bar.

**Conclusions:**

The molecular data suggest that some morphological characters of host-specific parasites may undergo convergent evolution in the divergent lineages, and therefore, to fully resolve the phylogenetic relationships among host-specific parasites, an integrative approach combining morphological and molecular data is still needed. In addition, our study indicates that parasite diversity in many regions is still under-explored, and thus we highlight the importance of studies of host-associated parasites, especially in the context of freshwater fish biogeography.

**Graphical Abstract:**

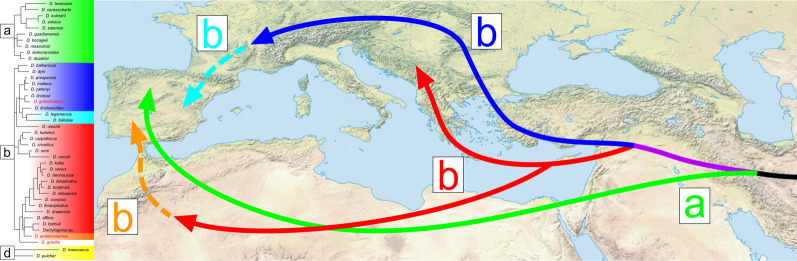

## Background

The geographical dispersion of host-specific parasites is strongly limited by the dispersal capabilities of their hosts. One of the most important events in the evolutionary history of parasites is cospeciation with their hosts, leading to the emergence of new species of parasites during the speciation of their hosts, which often occurs in allopatry [1]. However, such allopatric speciation of parasites may occur even prior to the divergence of their hosts, i.e., during the migration or translocation of hosts into new regions. Moreover, considering the faster life cycle of parasites compared to that of their hosts, parasites often tend to undergo sympatric speciation within a population of their respective hosts (usually referred to as intra-host duplication) [2–6].

One of the parasitic groups exhibiting a high degree of host specificity is ectoparasitic monogeneans parasitizing on fish. To attach themselves to gill apparatus, these parasites use a highly developed organ called the haptor. Monogeneans of the genus *Dactylogyrus* Diesing, 1850 are small-bodied common parasites of cyprinoid fishes (suborder Cyprinoidei, following the recent classification by [7]) [8–10]. *Dactylogyrus* is the most speciose genus among Platyhelminthes (more than 900 species, according to the last checklist compiled by [9]), which is most likely because of three major factors: the short developmental time of these parasites, the remarkably high host specificity in *Dactylogyrus*, and the numerousness of their common hosts. The haptor of *Dactylogyrus* parasites comprises one pair of anchor hooks, usually one dorsal and one ventral connective bar of various shapes, and seven pairs of marginal hooks (see [11] for morphotypes of haptoral sclerotized elements). Haptor morphometrics and the presence of specific sclerites apparently play an important role in the host specificity of *Dactylogyrus*, as the parasites of this genus require a high level of adaptation for the specific microhabitats provided by hosts ([12–14], reviewed in [10]). Some generalist species (e.g., *D. sphyrna, D. vastator*, and *D. vistulae*) have not developed a haptoral ventral connective bar. As this morphological feature is present in *Dactylogyrus* species of different phylogenetic lineages, it was hypothesized by Benovics et al. [15] that the secondary loss of the ventral connective bar may allow the parasite to infect a wider range of hosts. Concurrently, Šimková et al. [16] hypothesized that attachment organs with large-sized anchoral sclerites promote the colonization of several phylogenetically distant host species.

Following recent classification, Cyprinidae sensu stricto encompasses more than 1300 species [17] belonging to eleven morphologically diverse subfamilies (formerly tribes, as defined by [18]) with unequal distributions across Africa and Eurasia. Yang et al. [18] proposed five dispersion events to explain the present biogeographical distribution of these cyprinid lineages. At present, two subfamilies are recognized as native to the Afro- and Euro-Mediterranean: hexaploid, large sized Torinae (genera *Carasobarbus*, *Labeobarbus*, and *Pterocapoeta*) and highly diversified Barbinae (*Aulopyge*, *Barbus*, and *Luciobarbus*). Interestingly, while the phylogenetic relationships between *Barbus* and *Luciobarbus* are fully resolved and both genera are distinguishable on the basis of several autapomorphies (e.g., the number of pharyngeal teeth), *Luciobarbus* does not form a monophyletic group [18–22]. According to molecular phylogeny, the position of Middle Eastern *Capoeta* is nested within the lineage encompassing *Luciobarbus* species. Even though the diversity of cyprinids in the Middle East is not well documented (molecular data are especially scarce [23–25]), it appears that *Capoeta* represents the most speciose endemic genus [17]. Cyprinid fauna in the Middle East (Levant) is highly diversified in comparison to that in the Afro-Mediterranean and Euro-Mediterranean. The speciation of freshwater fauna was historically centered in the Mesopotamian Basin, where, before the Pliocene orogenesis, the Proto-Euphrates River maintained connection between the Black and Caspian Seas and allowed the mixing of African and Asian fish species [26, 27]. Therefore, the Middle East is considered to be a major biogeographical crossroad between biota of three continents, and the local fauna include cyprinoid genera also present in eastern Asia (e.g., *Garra* (Labeoninae) or *Schizothorax* (Schizothoracinae)) and Africa (i.e., *Luciobarbus* and *Carasobarbus*) [18, 28].

According to recent studies by Šimková et al. [29] and Benovics et al. [15, 30], endemic cyprinids of the peri-Mediterranean are parasitized by *Dactylogyrus* species belonging to at least three divergent phylogenetic lineages. The individual lineages are of uncertain origin and all aforementioned studies hypothesized their ancestry, which is most likely interconnected with the phylogeography and historical dispersion of their cyprinoid hosts. Reconstructions of *Dactylogyrus* phylogenies are commonly based on multiple molecular markers; such as partial genes coding ribosomal subunits (*18S, 28S, 5.8S*), and the internal transcribed spacer 1 (*ITS1*) [15, 16, 29–35]. The ribosomal subunits have been used to resolve phylogenetic relationships between divergent platyhelminth taxa for quite a long time (e.g. [36–39]) and represent slowly evolving parts of the genome under strong selection pressure. On the other hand, spliceosomal introns, such as ITS1, are generally composed of quasi-random sequences and due to their non-functional nature mutate much more rapidly over time (reviewed in [40]). The mechanisms of intron evolution are generally unknown; however, these regions are often used in the taxonomy of monogeneans, especially to investigate inter- and intraspecific variability (see [8, 30] for *Dactylogyrus* studies).

Since the majority of previous studies on *Dactylogyrus* phylogeny applied conservative markers for phylogenetic reconstruction (mainly due to aligning issues), it is tempting to assume that the inclusion of such variable non-coding segments may shed more light on the historical origin of *Dactylogyrus* parasites of cyprinids. Following the suggestion of Šimková et al. [29] that the Middle Eastern region may be considered a center of *Dactylogyrus* divergence predating the Messinian salinity crisis, the investigation of Middle Eastern endemic taxa may fill the gaps and resolve uncertain relationships between recent *Dactylogyrus* lineages. Unfortunately, prior to this study, the species diversity of Middle Eastern *Dactylogyrus* was largely under-explored and molecular data were missing. Thus, in the present study, we focused primarily on this region and, using an integrative approach combining molecular and morphological data, we investigated the phylogeography of *Dactylogyrus* parasites of peri-Mediterranean cyprinids. Accordingly, we discuss the potential scenarios of the dispersal of *Dactylogyrus* into Africa and Europe.

## Methods

### Collection and selection of *Dactylogyrus* species

For the purposes of this study, DNA sequences of cyprinid-specific *Dactylogyrus* species from Africa, Europe, and Eastern Asia were selected. The majority of sequences of partial genes coding small and large ribosomal subunits and the *ITS1* segment were retrieved from GenBank (hereinafter abbreviated as *18S*, *5.8S*, *28S*, and *ITS1*). In addition, new DNA sequences of the aforementioned genetic markers were obtained from *Dactylogyrus* species collected during the years 2018 and 2019 in Iran and Iraq, respectively (see Table 1 for accession numbers, host species, and countries of collection).

**Table 1:** List of investigated *Dactylogyrus* species in this study with countries and hosts of collection

*Dactylogyrus* species	*HS*	Ventral bar type	Region	Host	Subfamily	18S + ITS1	28S
*D. achmerowi*	4	Sphyrna	Eastern Asia	*Cyprinus carpio*	Cyprininae	MF683071	MF979966
*D. affinis*	3	Cornu	Middle East	*Barbus lacerta*	Barbinae	MZ031066*	MZ031054*
*D. anchoratus*	4	Sphyrna	Eastern Asia	*Carassius gibelio*	Cyprininae	KY859795	KY863555
*D. andalousiensis*	2	Rutili	Iberia	*Luciobarbus comizo*	Barbinae	MN365672	MN338207
*D. atlasensis*	1	Cornu	Morocco	*Luciobarbus lepineyi*	Barbinae	KY629337	KY629356
*D. balistae*	3	Sphyrna	Iberia	*Luciobarbus bocagei*	Barbinae	KY629344	MN338205
*D. balkanicus*	2	Wunderi	Europe	*Barbus prespensis*	Barbinae	KY201093	KY201107
*D. barbuli*	4	Cornu	Middle East	*Luciobarbus xanthopterus*	Barbinae	MZ031074*	MZ031063*
*D. benhoussai*	1	Cornu	Morocco	*Luciobarbus yahyaouii*	Barbinae	MN974254	MN973815
*D. bocageii*	3	Varicorhini	Iberia	*Luciobarbus graellsii*	Barbinae	MN365675	MN338210
*D. borjensis*	1	Cornu	Morocco	*Luciobarbus zayanensis*	Barbinae	MN974257	MN973819
*D. carassobarbi*	4	Varicorhini	Middle East	*Carasobarbus luteus*	Torinae	MZ031071*	MZ031060*
*D. carpathicus*	2	Cornu	Europe	*Barbus barbus*	Barbinae	KY201098	KY201111
*D. crivellius*	2	Cornu	Europe	*Barbus prespensis*	Barbinae	KY201094	KY201108
*D. doadrioi*	2	Varicorhini	Iberia	*Luciobarbus guiraonis*	Barbinae	MN365682	KY629346
*D. draaensis*	1	Cornu	Morocco	*Luciobarbus lepineyi*	Barbinae	MN974258	MN973816
*D. dyki*	2	Wunderi	Europe	*Barbus barbus*	Barbinae	KY629338	KY629367
*D. falciformis*	4	Sphyrna	Eastern Asia	*Cyprinus carpio*	Cyprininae	MZ031072*	MZ031061*
*D. falsiphallus*	1	Cornu	Morocco	*Luciobarbus maghrebensis*	Barbinae	MN974253	MN973813
*D. fimbriphallus*	2	Cornu	Morocco	*Luciobarbus lepineyi*	Barbinae	KY629332	KY629357
*D. formosus*	2	Sphyrna	Eastern Asia	*Carassius gibelio*	Cyprininae	MG792869	MG792984
*D. goktschaicus*	3	Rutili	Middle East	*Barbus lacerta*	Barbinae	MZ031067*	MZ031055*
*D. gracilis*	3	Rutili	Middle East	*Capoeta buhsei*	Barbinae	MZ031068*	MZ031056*
*D. guadianensis*	2	Varicorhini	Iberia	*Luciobarbus comizo*	Barbinae	MN365674	MN338209
*D. inexpectatus*	2	Sphyrna	Eastern Asia	*Carassius gibelio*	Cyprininae	AJ564138	AJ969945
*D. ksibii*	2	Cornu	Morocco	*Luciobarbus ksibi*	Barbinae	MN974252	MN973812
*D. kulindrii*	2	Varicorhini	Morocco	*Carasobarbus fritschii*	Torinae	KY629336	KY629354
*D. kulwieci*	4	Cornu	Middle East	*Luciobarbus xanthopterus*	Barbinae	MZ031075*	MZ031064*
*D. legionensis*	2	Sphyrna	Iberia	*Luciobarbus graellsii*	Barbinae	MN365678	MN338213
*D. lenkorani*	4	Varicorhini	Middle East	*Capoeta buhsei*	Barbinae	MZ031069*	MZ031057*
*D. lenkoranoïdes*	3	Varicorhini	Iberia	*Luciobarbus graellsii*	Barbinae	MN365676	MN338211
*D. linstowi*	4	Wunderi	Middle East	*Luciobarbus capito*	Barbinae	MZ031073*	MZ031062*
*D. linstowoïdes*	2	Wunderi	Iberia	*Luciobarbus guiraonis*	Barbinae	KY629329	KY629349
*D. malleus*	2	Wunderi	Europe	*Barbus barbus*	Barbinae	KY201099	KY201112
*D. marocanus*	4	Magnihamatus	Morocco	*Carasobarbus fritschii*	Torinae	KY629333	KY629355
*D. mascomai*	3	Varicorhini	Iberia	*Luciobarbus graellsii*	Barbinae	MN365680	MN338215
*D. omenti*	3	Cornu	Europe	*Aulopyge huegelii*	Barbinae	KY201091	KY201105
*D. petenyi*	2	Wunderi	Europe	*Barbus balcanicus*	Barbinae	KY201097	KY201113
*D. prespensis*	1	Wunderi	Europe	*Barbus prespensis*	Barbinae	KY201096	KY201110
*D. pulcher*	4	Magnihamatus	Middle East	*Capoeta capoeta*	Barbinae	MZ031070*	MZ031058*
*D. remi*	1	Cornu	Europe	*Luciobarbus graecus*	Barbinae	KY201101	KY201115
*D. romuli*	1	Cornu	Europe	*Luciobarbus albanicus*	Barbinae	KY201100	KY201114
*D. scorpius*	1	Cornu	Morocco	*Luciobarbus rifensis*	Barbinae	MN974256	MN973818
*D. varius*	1	Cornu	Morocco	*Luciobarbus maghrebensis*	Barbinae	MN974255	MN973814
*D. vastator*	4	Sphyrna	Eastern Asia	*Carassius gibelio*	Cyprininae	KY207446	MZ031059*
*D. volutus*	1	Varicorhini	Morocco	*Carasobarbus fritschii*	Torinae	KY629334	KY629353
*D. zatensis*	1	Varicorhini	Morocco	*Carasobarbus fritschii*	Torinae	KY629335	KY629352
*Dactylogyrus* sp.	3	Cornu	Middle East	*Luciobarbus xanthopterus*	Barbinae	MZ031076*	MZ031065*

HS = level of host specificity: (1) strict specialists, (2) intermediate specialists, (3) transitional generalists, (4) common generalists; *18S* rDNA plus *ITS1* and *28S* rDNA = GenBank accession numbers of sequences of respective genetic loci. Newly generated sequences in this study are marked by asterisks (*)

In the field, *Dactylogyrus* specimens were removed from the gills of fish hosts during standard parasitological dissection (according to [41]). The majority of specimens were mounted on a slide, covered with a coverslip, and fixed in a mixture of glycerine and ammonium picrate (GAP [42]) in order to expose taxonomically important morphological characters. At least five specimens of each newly collected *Dactylogyrus* species were selected and cut into two parts using fine needles, one half (usually the one with the copulatory organs) mounted on a slide and fixed for further morphological evaluation, the other half (usually the one with the haptor) fixed in 96% pure ethanol for the subsequent isolation of DNA. The sclerotized parts of the haptor (i.e., haptoral sclerites) and the reproductive organs (male copulatory organ and vaginal armament) were used for species determination, following Pugachev et al. [11]. Identification at species level was performed using an Olympus BX51 microscope (Olympus, Tokyo, Japan) equipped with phase-contrast optics.

### DNA extraction, amplification, and sequencing

Bisected *Dactylogyrus* preserved in ethanol were dried using a vacuum centrifuge. The extraction of whole genomic DNA was performed using DNEasy Blood & Tissue Kit (Qiagen, Hilden, Germany) following the protocol provided by the manufacturer. Up to four genetic markers were used for *Dactylogyrus*. The partial *18S*, entire *ITS1*, and partial *5.8S* regions were amplified using the primers S1 (forward, 5′-ATTCCGATAACGAACGAGACT-3′) and Lig5.8R (reverse, 5′-GATACTCGAGCCGAGTGATCC-3′), which anneal to the segments of DNA coding *18S* and.

*5.8S*, respectively [43, 44]. Amplification reactions followed protocols optimized in Benovics et al. [15]. The partial *28S* region was amplified using the forward primer C1 (5′-ACCCGCTGAATTTAAGCA-3′) and reverse primer D2 (5′- TGGTCCGTGTTTCAAGAC-3′) [45], following the PCR protocol optimized by Šimková et al. [34]. The PCR products (~ 1000 bp for *18S*, *ITS1*, and *5.8S*, and ~ 800 bp for partial *28S*) were checked on 1% agarose gel and purified using the ExoSAP-IT kit (Amplia, Bratislava, Slovakia) following the standard protocol. The purified products were directly sequenced using the same primers as for PCR and BigDye Terminator Cycle Sequencing kit (Applied Biosystems, Foster City, USA). Sequencing was performed on an ABI 3130 Genetic Analyzer (Applied Biosystems, Foster City, USA).

### Phylogenetic analyses and the mapping of the characters

A DNA sequence alignment including 49 sequences, i.e., 48 *Dactylogyrus* species and one outgroup taxon *Ancyrocephalus percae*, was constructed by concatenating partial genes for *18S* and *28S* rRNA, and the *ITS1* region. Homolog sequences were aligned using the Fast Fourier transform algorithm in MAFFT [46] and ends were manually trimmed to unify their length. The data were treated as partitioned, and GTR (the general time-reversible evolutionary model) was applied for each partition. The shape parameter of the gamma distribution (G) and the proportion of invariable sites (I) were selected for each gene segment individually using PartitionFinder v.2 [47, 48]. Phylogenetic analyses using maximum likelihood (ML) were computed employing RaxML v.8.1.12 [49, 50]. The best ML tree was selected from 1000 iterations, and support for the branching pattern was validated through 5 × 10^3^ pseudoreplicates. Phylogenetic analyses of Bayesian inference (BI) were carried out in MrBayes v.3.2 [51], and the resulting tree was constructed using the Metropolis-coupled Markov chain Monte Carlo algorithm. Four concurrent chains (one cold and three heated) ran for 5 × 10^7^ generations, sampling trees every 10^2^ generations. The first 30% of trees were discarded as a relative burn-in period after checking that the standard deviation split frequency fell below 0.01. Results were checked in Tracer v.1.7.1 [52] to assess convergence. Posterior probabilities were calculated as the frequency of samples recovering particular clades.

The mapping of morphological characters onto the phylogenetic tree containing all investigated parasite species (resulting from the first phylogenetic analysis) was performed in Mesquite v.3.2 [53]. The character mapped onto the phylogenetic tree was the haptoral ventral connective bar, representing the most variable morphological character in *Dactylogyrus*, ranging from well-developed with five extremities up to completely absent (see [11, 16] for morphotypes).

### Levels of host specificity in *Dactylogyrus* parasites

Considering the delimitation of host specificity for *Dactylogyrus* by Šimková et al. [16] and taking into account the present classification of cyprinoids [7], the modified version of levels of host specificity is presented in Table 1. The *Dactylogyrus* species were divided into four categories: (1) strict specialists parasitizing single cyprinoid species, (2) intermediate specialists parasitizing congeneric host species, (3) transitional generalists parasitizing cyprinoid species belonging to a single subfamily, and (4) common generalists parasitizing species belonging to a single cyprinoid family, i.e., in this case, to Cyprinidae. The level of host specificity for each investigated *Dactylogyrus* species was determined from the compilation of data from various sources, i.e., checklists ([9, 54–57] and references within), the determination key including original descriptions and/or host reports included in Pugachev et al. [11], and other studies reporting the presence of the investigated species [8, 15, 30–32, 35, 58–69]. All published reports were thoroughly evaluated, and potential misidentifications were not included. Publicly unavailable and/or non-traceable reports (e.g., master’s or Ph.D. theses) were not taken into account.

## Results

### Host specificity in *Dactylogyrus* of Cyprinidae

The list of investigated *Dactylogyrus* species with their levels of host specificity is presented in Table 1. From 48 *Dactylogyrus* species, 12 species belonged to strict specialists, specific to a single host species. The majority of species with this narrow host specificity were from Northwest Africa. Sixteen species were intermediate specialists, i.e., parasitizing only cyprinid host species belonging to a single genus. Nine species were classified as transitional generalists parasitizing hosts from at least two different genera belonging to a single cyprinid subfamily (i.e., Barbinae, Cyprininae, or Torinae). Finally, eleven species were recognized as common generalists, i.e., species parasitizing multiple host species belonging to different subfamilies (within Cyprinidae).

### Phylogenetic position of Middle Eastern *Dactylogyrus* species

**Fig. 1:**
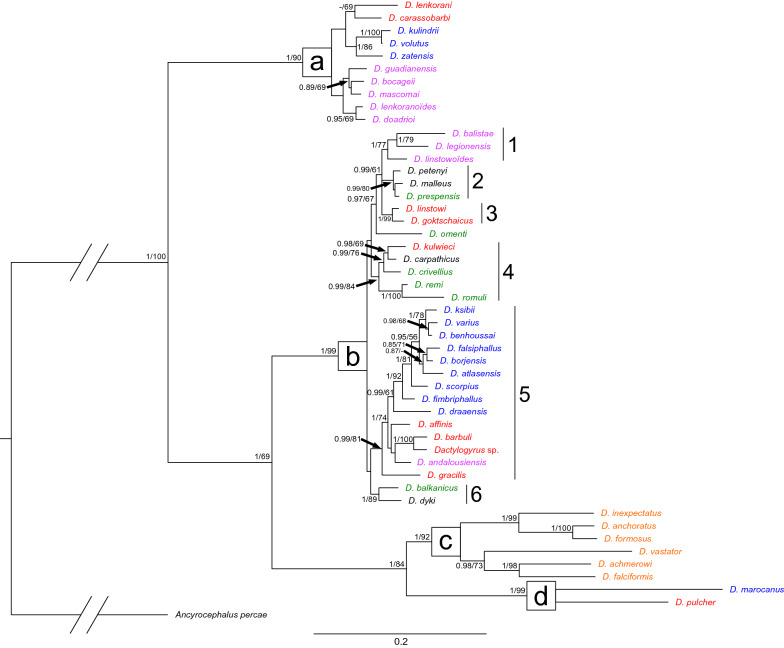
Phylogenetic tree of 48 *Dactylogyrus* species from cyprinids resulting from BI analysis. The tree is based on concatenated partial sequences of genes coding *18S* rRNA and *28S* rRNA, and the *ITS1* region. Numbers along branches represent posterior probabilities (> 0.80) and bootstrap support values (> 50) for individual nodes, resulting from BI and ML analyses, respectively. Lower values are shown as dashes (–). The length of branches represents the number of substitutions per site. The letters (**a**–**d**) and the numbers (1–6) represent specific clades. The colours represent regions of distribution of the respective *Dactylogyrus* species: blue—Northwest Africa; red—Middle East; violet—Iberian Peninsula; green—Balkan and Apennine Peninsulas; black—various regions across Europe; orange species are common parasites of cyprinins originating from Eastern Asia

The final alignment for phylogenetic reconstruction spanned 2075 nucleotide positions (445 positions for the partial gene coding *18S* rRNA, 699 positions for the partial gene coding *28S* rRNA, and 931 bp positions for the partial *ITS1* region). The ML and BI analyses generated trees with congruent topologies. Therefore, only the BI tree is presented (Fig. 1), with values along nodes indicating posterior probabilities resulting from BI, and bootstrap support from ML analyses. The investigated *Dactylogyrus* species formed four major well-supported clades. The first clade (a) encompassed two Middle Eastern species (i.e., *D. lenkorani* and *D. carassobarbi*), three *Dactylogyrus* species host specific to North African *Carasobarbus* (i.e., *D. kulindrii, D. volutus*, and *D. zatensis*), and five species host specific to endemic *Luciobarbus* spp. of the Iberian Peninsula. The second clade (b) encompassed all *Dactylogyrus* species parasitizing European *Barbus* spp. and the Balkan *Luciobarbus* and *Aulopyge*, four species parasitizing endemic Iberian *Luciobarbus* spp., the majority of *Dactylogyrus* species parasitizing North African cyprinids, and eight Middle Eastern *Dactylogyrus* species. Six well- or moderately supported monophyletic groups were revealed within clade b, even though not all phylogenetic relationships between these groups were fully resolved by either BI or ML analysis. The phylogenetic analyses strongly support the phylogenetic proximity of Middle Eastern *D. goktschaicus* and *D. linstowi* (group b3); however, their positions within clade b and their relationships to other groups were not resolved. The other two well-supported groups within clade b include three species of Iberian *Luciobarbus* spp. (i.e., *D. balistae, D. legionensis,* and *D. linstowoïdes*; group b1), and three species of Central European *Barbus* spp. (i.e., *D. petenyi, D. prespensis,* and *D. malleus*; group b2), respectively. Similarly to group b3 including two Middle Eastern species, the phylogenetic position of these two lineages within clade b was also not resolved. However, the basal position of *D. omenti* of the endemic Balkan *A. huegelii* to these three lineages (i.e., b1–3) was well/moderately supported. Another well-supported group encompasses four European *Dactylogyrus* species parasitizing Balkan *Luciobarbus* spp. (*D. romuli* and *D. remi*) and *Barbus* spp. (*D. carpathicus* and *D. crivellius*), and *D. kulwieci* parasitizing Middle Eastern *Luciobarbus xanthopterus* (group b4). Also, the monophyly of *Dactylogyrus* species of North African cyprinids (blue species in group b4) was well supported by both analyses. Four Middle Eastern species (i.e., *D. affinis, D. gracilis, D. barbuli*, and *Dactylogyrus* sp.) and *D. andalousiensis* (a species endemic to the Iberian Peninsula) were revealed as phylogenetically close to the North African lineage. The last well-supported group was formed by two common *Dactylogyrus* spp. of *Barbus* spp.—specifically, *D. balkanicus* and *D. dyki* (group b6). Even though the monophyly of these two species was strongly supported by both phylogenetic analyses, neither analysis resolved their position within clade b. Clade c included all investigated *Dactylogyrus* species specific to *Cyprinus carpio* and *Carassius gibelio* (putative of an East Asian origin)*.* The last well-supported clade (d) included only two species—*D. marocanus* of North African *Carasobarbus fritschii,* and *D. pulcher* of Middle Eastern *Capoeta* spp.

### Diversity of haptoral elements in *Dactylogyrus* of Cyprinidae

**Fig. 2:**
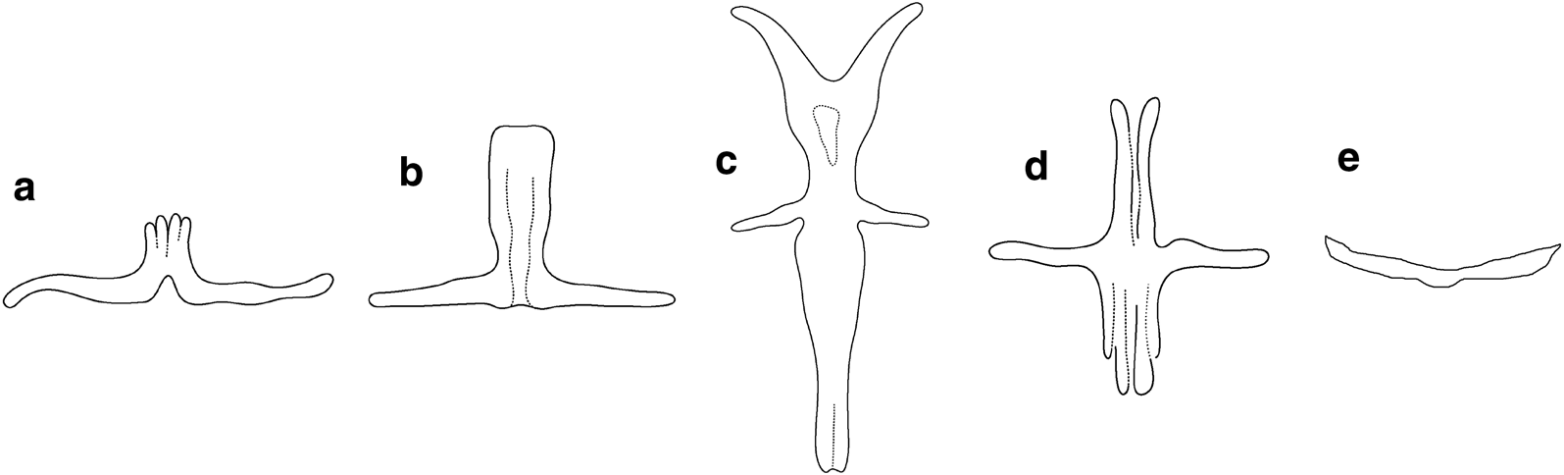
Morphological types of the haptoral ventral connective bars of *Dactylogyrus* parasites in this study. **a** “varicorhini” type, **b** “wunderi” type, **c** “cornu” type, **d** “rutili” type, **e** “magnihamatus” type

**Fig. 3:**
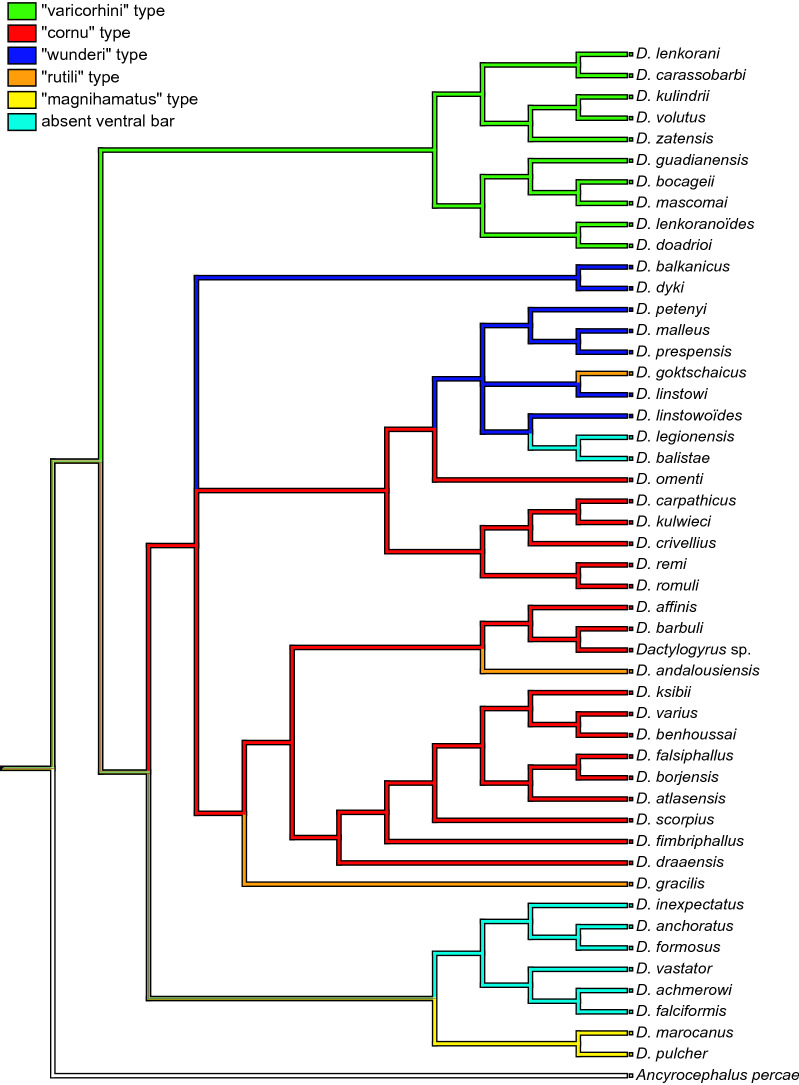
Mapping of the morphological types of haptoral ventral bars into *Dactylogyrus* phylogeny performed in Mesquite. The specific colors are corresponding to morphological types in the Fig. 2

Out of the 17 basic morphological types of haptoral ventral bar (taken from [11]), five were present in the *Dactylogyrus* species of peri-Mediterranean cyprinids: the “cornu” double-cross-shaped type with five projections; the “rutili” cross-shaped with four projections; the “wunderi” three-armed triangular type; the “varicorhini” almost linear shape with V-shaped tubercle in the central part; and the small “magnihamatus” linear type (Fig. 2). A mapping of the morphological types of haptoral ventral connective bars into *Dactylogyrus* phylogeny is presented in Fig. 3. Six *Dactylogyrus* species parasitizing *C. carpio* and *C. gibelio,* and two *Dactylogyrus* species endemic to Iberian Peninsula (i.e., *D. balistae* and *D. legionensis*) had no ventral bar (clades c and d in Fig. 1). The most common type of ventral bar among the investigated species was the “cornu” type possessed by all *Dactylogyrus* species parasitizing Northwest African and Balkan *Luciobarbus* spp., two intermediate specialist species parasitizing European *Barbus* spp., *D. omenti*, and four Middle Eastern *Dactylogyrus* species (i.e. all species of subclade b4 plus *D. omenti* and the majority of species of subclade b5). The “varicorhini” type of ventral bar was possessed by 10 *Dactylogyrus* species, all belonging to clade A. The “wunderi” type of ventral bar was the most prevalent among the *Dactylogyrus* of European *Barbus* spp. (possessed by 2 species of clade b6 and 3 species of clade b2). This type of ventral bar is also present in Middle Eastern *D. linstowi* and Iberian *D. linstowoïdes.* The *“*rutili*”* type was only present in *D. gracilis, D. andalousiensis,* and *D. goktschaicus*, even though in the last species this bar visibly differed in the width and shape of its projections compared to the former two species. The strongly miniaturized ventral bar of the “magnihamatus” type, in the shape of a thin line, was possessed by *D. marocanus* and *D. pulcher—*within our study, the only representatives of clade d.

## Discussion

The present study was focused on the host specificity, phylogenetic relationships, and morphological diversity of *Dactylogyrus* parasites of Cyprinidae in three major regions in the peri-Mediterranean—North Africa, Southern Europe, and the Middle East. The Mediterranean is characterized by high diversity, especially that of endemic cyprinoid species, and encompasses several highly diversified, phylogenetic lineages of cyprinoids, each with a putatively different historical origin [18, 22, 70–74]. The phylogenetic relationships between *Dactylogyrus* parasites of North African and European cyprinoids were previously investigated over a relatively wide geographical scale by Šimková et al. [29] and Benovics et al. [15]. Even though both studies concluded that *Dactylogyrus* parasites, like their cyprinid hosts, are of multiple historical origins, there are still some uncertain relationships between present lineages of *Dactylogyrus*, and the inclusion of Middle Eastern species into phylogenetic reconstruction may shed more light into these uncertainties. Unfortunately, prior to the present study, publicly available molecular data on Middle Eastern *Dactylogyrus* species were almost nonexistent.

Compiling the data from 48 peri-Mediterranean *Dactylogyrus* species revealed that 25% of them are strict specialists, associated with only a single cyprinid host species. Nine of 12 strictly specific species are parasites of Northwest African Barbinae and Torinae (i.e., *Luciobarbus* spp. and *Carasobarbus fritschii,* respectively). Of the other three strictly specific species, two are associated with one of two Balkan *Luciobarbus* spp., and *D. prespensis* is specific to *Barbus prespensis.* Both Balkan *Luciobarbus* spp. and *B. prespensis* are endemic to the Balkan Peninsula [75] and exhibit a limited distribution range. Therefore, we can assume that their respective host-specific parasite species cospeciated with their hosts after diversification in the respective regions. To date, only three works have been published investigating the diversity of endemic *Dactylogyrus* in the Afro-Mediterranean, these describing new *Dactylogyrus* species from North African cyprinids [65, 73, 76]. A total of 17 species are known from the Afro-Mediterranean and apparently more than half are strictly host specific. However, since data from this region are still scarce, as all three works are only from Morocco or Algeria, we can only assume that the diversity of *Dactylogyrus* is much higher than so far reported in the Afro-Mediterranean. Moreover, this may also imply that endemic hosts are under-explored and, therefore, that the host range of some endemic *Dactylogyrus* species may be wider than is actually documented, similarly as for *D. omenti.* This species was recently considered as a specialist of *A. huegelii* in the Balkans [31]; however, Koyee and Abdullah [62] reported *D. omenti* also from Middle Eastern *L. xanthopterus.* The validity of *D. omenti* was in their study also supported by the molecular data. In the present study, 33% of investigated species were reported as intermediate specialists, i.e., parasitizing congeneric host species. These species appear to be the most common in Europe, where the distribution ranges of congeneric cyprinids from two highly divergent genera (i.e., *Barbus* and *Luciobarbus*) often overlap. Therefore, *Dactylogyrus* parasites, due to their biological preconditions for host-switching (e.g., a free-living larval stage, or a sympatric distribution of phylogenetically related host species, further discussed in [15, 32]) are more likely to be present on two phylogenetically closely related hosts. The fraction of intermediate (congeneric) specialists in the peri-Mediterranean is higher in comparison to literature data compiled by Kuchta et al. [77], who reported that in *Dactylogyrus* parasitizing European cyprinoids strict specificity is less frequent than intermediate specificity. However, it is important to note that Leuciscidae (*Dactylogyrus* species diversity in all cyprinoids, including Leuciscidae, was analyzed by Kuchta et al. [77]) represent a far more diversified cyprinoid family in comparison to Cyprinidae [7, 75]. Highly diversified leuciscid taxa, whose distribution in the peri-Mediterranean is usually limited to a single river or lake system [75], more likely harbour endemic host-specific *Dactylogyrus* species [8, 30, 31, 78–80]. In our study, *Dactylogyrus* species parasitizing hosts belonging to phylogenetically close genera (i.e., genera from a single subfamily) represent 19% of *Dactylogyrus* species in the peri-Mediterranean. These species are especially common in the Middle East, which is the most likely related to the distribution of the divergent cyprinid genera (*Barbus*, *Luciobarbus*, *Capoeta*, and *Carasobarbus*) in this region. Since only three cyprinid genera besides *Cyprinus* and *Carassius* are natively present in Europe (monotypic *Aulopyge,* highly diversified *Barbus,* and *Luciobarbus*—all three belonging to Barbinae) and their areas only rarely overlap, the possibilities for host-switching between species of different genera are limited and, therefore, rather rare. A notable exception is the northern part of the Iberian Peninsula belonging to the distribution range of southwest European endemic *Barbus meridionalis*, which often hybridizes with endemic Iberian *B. haasi* [81]. These *Barbus* species live on the Peninsula in sympatry with endemic *Luciobarbus* spp. [75, 82, 83], which putatively facilitates the host-switching of parasites between non-congeners. Hybridization between Iberian *Luciobarbus* and *Barbus* (suggested by [83]) may be another factor further promoting the intergeneric host-switching of parasites. Thus, in Iberia, *Dactylogyrus* species common to Central European *Barbus* spp. are almost absent, and *Dactylogyrus* fauna of endemic *B. haasi* was completely replaced by endemic *Dactylogyrus* species which are typical for *Luciobarbus* spp. in the region (e.g., *D. balistae*, *D. bocageii*, or *D. mascomai*) [30, 84]. However, this one-way drift of parasite fauna has not yet been explained. The situation is different in the Middle East, where, in addition to *Barbus* and *Luciobarbus*, the presence of endemic genera is documented, i.e., *Capoeta* (phylogenetically closely related to Euro-Mediterranean *Luciobarbus* [20, 22, 85]), and *Cyprinion, Scaphiodonichthys* and *Semiplotus* (genera in the basal position to all other peri-Mediterranean Barbinae [18]). Therefore, the intergeneric host-switching of parasites in this region, where overlapping habitats of non-congeneric cyprinids are more common [23, 25, 86], is also reflected in the observed host specificity of endemic *Dactylogyrus* parasites.

Endemic generalist *Dactylogyrus* species were almost exclusively present on cyprinids in the Middle East (the exception was North African *D. marocanus*)—a region where the distribution ranges of native Barbinae and Torinae overlap. However, a slightly different trend is observable in multiple European regions—species, presumably specialists on representatives of Cyprininae (e.g. *Cyprinus* or *Carassius*), have been introduced together with their hosts into non-native regions in the Euro-Mediterranean, and, according to recent reports, have spread to endemic non-congeneric cyprinid hosts (such as *D. achmerowi* and *D. falciformis* on *B. plebejus* in the Apennine Peninsula [60], or *D. vastator* on *A. huegelii* in the Balkans [31], and *B. plebejus* in the Apennine Peninsula [60]), resulting in an increased host range.

The host range of an undetermined *Dactylogyrus* sp. collected from Iraq *L. xanthopterus* remains unknown, as this species is potentially new to science. Comparing only morphological data, Raissy and Ansari [66] reported specimens of this species from *B. barbulus* in Iran, which was in their study identified as *D. akaraicus*. Nonetheless, the species reported by the authors does not morphologically match the original description by Mikailov [87], and therefore we concluded that the species in their study was misidentified. Despite this, we can still assume that the host range of *Dactylogyrus* sp. encompasses species of at least two genera (belonging to Barbinae), and thus this *Dactylogyrus* species was categorized as an intermediate generalist.

In previous studies, Šimková et al. [29] and Benovics et al. [15] discussed the phylogenetic origin of the host-specific monogeneans of cyprinids. Both studies concluded that *Dactylogyrus* species parasitizing peri-Mediterranean cyprinids form three divergent phylogenetic lineages. Moreover, they also suggested congruently that the addition of Middle Eastern congeners into phylogenetic analyses of *Dactylogyrus* may shed more light into the phylogenetic relationships of these host-specific parasites, because the Middle East represents a putative region of the ancestral diversification of cyprinoids prior to their dispersion into Europe and Africa [18, 72, 88–92]. Following previous suggestions, herein we present the first phylogenetic reconstruction of *Dactylogyrus* including also molecular data on *Dactylogyrus* species parasitizing Middle Eastern cyprinids. Three previously revealed phylogenetic lineages are congruent with clades a, b, and c in our present study. In addition, we also propose a fourth lineage formed by *D. marocanus* and *D. pulcher,* in sister position to the clade of *Dactylogyrus* of *Cyprinus* and *Carassius.* Šimková et al. [29] suggested that this lineage should also include *Dactylogyrus* species of West African cyprinids (e.g., *Dactylogyrus* species parasitizing *Labeo* hosts). Unfortunately, multiloci molecular data for these species, described from labeonins in Senegal [93–95], are not yet publicly available. Since for the phylogenetic reconstruction in the present study we combined slowly evolving conservative regions (ribosomal subunits) with the rapidly evolving non-coding region (ITS1) in order to add more information to resolve the phylogenetic relationships between lineages within major clades, African *Dactylogyrus* species were omitted from the final dataset; therefore, we can only assume such phylogenetic relatedness from the previous work of Šimková et al. [29].

The monophyly of *D. pulcher* and *D. marocanus* is also supported by morphology; both species possess a haptoral ventral bar of the “magnihamatus” type and anchor hooks with elongated inner roots (see [11] for morphology). The same morphological features are also shared by the *Dactylogyrus* species parasitizing West African *Labeo* (see [95, 96]), further supporting the phylogenetic proximity of *D. pulcher* and *D. marocanus* to congeners of widely distributed African cyprinids of *Labeo*. In addition, the similarity in haptoral features, especially the absence of a ventral connective bar, is also recognized in the *Dactylogyrus* of cyprinins of presumed West-Asian origin (e.g. *D. inexpectatus*, *D. anchoratus*, or *D. formosus* parasitizing *C. carpio* and/or *C. gibelio*). Therefore, considering the molecular data and the morphology of haptoral elements, which according to Šimková et al. [16], Vignon et al. [97], and Benovics et al. [15] are the optimal characters for resolving the phylogeny of monogenean parasites (especially *Dactylogyrus*), we can assume that species from clades c and d, and *Dactylogyrus* of African labeonins share a common ancestry with their hosts in eastern Asia. From all the above, we hypothesize that species of clade d (together with *Dactylogyrus* of African Labeonins) are descendants of the first colonization wave of *Dactylogyrus* into Africa occurring via the Gomphotherium land bridge around 19 MYA [90, 98–100]. This hypothesis is also supported by the discovery of the oldest *Labeo*-like fossils in Africa, which were found to be approximately 17 million years old [101].

**Fig. 4:**
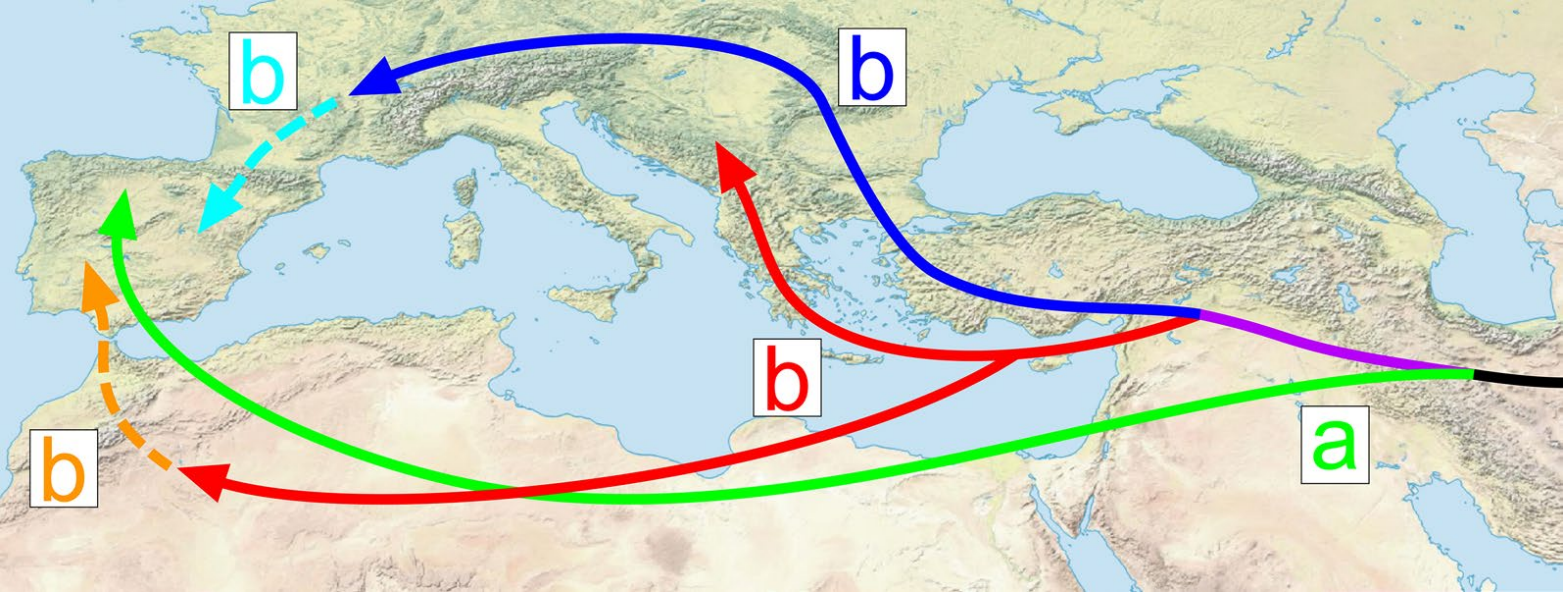
Hypothesized historical dispersion routes of peri-Mediterranean *Dactylogyrus* species. The colors of lines, arrows, and letters correspond to the colors for lineages in Fig. 3

According to our phylogenetic reconstruction, all investigated *Dactylogyrus* of Middle Eastern cyprinids belong to three phylogenetic clades (a, b, and d). The monophyly of the species within clade a is supported by both molecular data and morphology. The three Northwest African species of *C. fritschii* and five Iberian species clustered with Middle Eastern *D. lenkorani* and *D. carassobarbi*—all species possessing the “varicorhini” type of haptoral connective ventral bar. The majority of the species within clade a do not exhibit a high level of host specificity. Even though African species parasitize only fish belonging to hexaploid Torinae (*Carasobarbus* and *Labeobarbus* [73]) and Iberian species parasitize only fish from tetraploid Barbinae (*Barbus* and *Luciobarbus* [30, 84], Middle Eastern *Dactylogyrus* species have been reported from fish of both subfamilies (e.g. [55]). Due to the presence of *Dactylogyrus* parasitizing *Carasobarbus* and *Capoeta* within clade a, we can hypothesize that the historical origin of the species is associated with the historical dispersion of the common ancestor of large hexaploid torins (e.g., *Labeobarbus*, *Carasobarbus*, and *Pterocapoeta*) into Africa (route a, Fig. 4), after their former hexaploidization and divergence from the tetraploid lineage, which occurred in Western Asia during the Miocene [18, 85].

Considering the host range of *Dactylogyrus* species, we can assume that in contrast to the previously mentioned clade a, the historical origin of species belonging to clade b is associated with the dispersion of the Barbinae into Europe and North Africa (route b, Fig. 4). This clade encompasses the rest of the investigated Middle Eastern *Dactylogyrus* species, the majority of *Dactylogyrus* of Northwest African cyprinids (*Luciobarbus*), and all *Dactylogyrus* species parasitizing European *Barbus* spp. and *A. huegelii*, and six species of European *Luciobarbus* spp. (including four Iberian species and two Balkan species). While the morphology regarding the haptoral elements mostly supported the phylogenetic position of each individual species within the previous clades (a, c, and d), the species within clade b exhibit rather higher diversity in the attachment apparatus. The monophyly of all African species of clade b was well supported—these species include all specialists parasitizing *Luciobarbus* and possessing a haptoral ventral bar of the “cornu” type (see [65, 73, 76] for morphology). In the basal position to this clade were three *Dactylogyrus* species of Middle Eastern cyprinids (namely, *D. affinis*, *D. barbuli,* and *Dactylogyrus* sp.), and *D. andalousiensis* of Iberian *Luciobarbus* spp. Even though the phylogenetic relationships between these basal Middle Eastern species were not fully resolved, their morphological similarities support their phylogenetic proximity to the Northwest African *Dactylogyrus* species of *Luciobarbus* (i.e., all possessing the connective ventral bar of the “cornu” type; for morphology, see the original descriptions by Bychowsky [102] and Gussev et al. [103], respectively, or [11]). The same type of connective ventral bar is recognized in the species from lineage b4, which encompasses *D. remi* and *D. romuli* of endemic Balkan *Luciobarbus,* two species parasitizing European *Barbus*, and *D. kulwieci* parasitizing Middle Eastern cyprinids. In addition to similarities in the haptoral elements, all these five species possess copulatory organs of similar shape, categorized by Řehulková et al. [67] as the type of the “Caspian” group (as opposed to the type of the “Moroccan” group possessed by *Dactylogyrus* of Northwest African and Middle Eastern cyprinids from clade b5). The presence of the “cornu” type of haptoral ventral bar in species of two *Dactylogyrus* phylogenetic lineages indicates historical divergence and two individual dispersion routes of an ancestor possessing this type of ventral bar into the peri-Mediterranean (red route b, Fig. 4). Other *Dactylogyrus* parasitizing European *Barbus* (i.e., lineages b2 and b6) possess a haptoral ventral bar of the “wunderi” type (see [11] for morphology), and therefore we can assume that they are also phylogenetically close, even though the molecular data did not support this. The same type of haptoral bar is also recognized in *D. linstowi* parasitizing Middle Eastern cyprinids. However, even though *D. goktschaicus* is, according to the phylogenetic reconstruction, close to *D. linstowi*, it possesses a haptoral ventral bar more resembling the “rutili” type, which is more common in the *Dactylogyrus* parasitizing leuciscids, and, in our dataset, also possessed by Middle Eastern *D. gracilis* and Iberian *D. andalousiensis* from clade b5 [11]. Nevertheless, there are measurable differences in the sclerotized parts of the haptor of *D. goktschaicus* in comparison to the latter two species from clade b5: (1) the ventral bar is, in general, larger in respect to other haptoral sclerites, and (2) the anterior and posterior projections of the ventral bar are comparatively wider in respect to the ventral bars of the other two species. Therefore, we can postulate two hypotheses. The first is that the haptoral ventral bar of the “rutili” type evolved convergently in *Dactylogyrus* parasitizing cyprinids, as the rutili” type may represent a form of the ventral bar derived from either the “cornu” type by miniaturization of the posterior and anterior projections, or the “wunderi” type by enlargement of the posterior projection and the bifurcation of the anterior projection. The convergent evolution revealed by similarities in the haptoral elements recognized in some *Dactylogyrus* species of the two lineages of clade b is potentially also supported by Benovics et al. [15], who suggested two different historical dispersion routes for *Dactylogyrus* species in clade b—the southern route for species of clades b4 and b5 associated with the dispersions of North African *Luciobarbus* and Balkan cyprinids (red routes b, Fig. 4), and the northern route for species of clades b1, b2, and b6 via Europe with ancestral *Barbus* hosts (blue route, Fig. 4) (proposed by [72]). The phylogenetic position of *D. andalousiensis*, which also possesses a ventral bar resembling the “rutili” type, but is endemic to the Iberian Peninsula, also supports the first hypothesis, i.e., the ventral bar evolved convergently in the Iberian Peninsula (illustrated by the orange route in Fig. 4). The second hypothesis is that the “rutili” type is, in fact, the ancestral state of the ventral bar for *Dactylogyrus* of clade b, and that the two derived types, i.e., “cornu” and “wunderi,” developed after the divergence from common ancestor, and during the historical dispersion of the two divergent Barbinae host lineages [72, 89]. In this case, regarding morphology, *D. andalousiensis* represents a slowly evolving species.

## Conclusion

Fish of the highly diversified taxon Cyprinidae harbor a remarkably species-rich group of host-specific *Dactylogyrus* parasites. In general, the phylogenetic relationships between present *Dactylogyrus* lineages are concurrent with the phylogeny of their associated host lineages and are shaped by the historical dispersion of cyprinids in the peri-Mediterranean. However, the distribution of the *Dactylogyrus* lineages is influenced more by the diversity of divergent host lineages in the respective regions. The association of individual *Dactylogyrus* species (or lineages) with a particular dispersal event proposed for cyprinids may often be recognized at first sight by the morphological characters of the parasite attachment organ. However, the molecular data suggest that some morphological characters of host-specific parasites may undergo convergent evolution in the divergent lineages; therefore, to fully resolve the phylogenetic relationships among host-specific parasites, an integrative approach combining morphology and molecular data is still needed. Lastly, our study clearly indicates that parasite diversity in some biogeographical regions of fish distribution is still under-explored, and therefore we highlight the importance of studies of host-associated parasites, especially in the context of freshwater fish biogeography.

## Data Availability

All new sequences of *Dactylogyrus* obtained for the purposes of this study were deposited in NCBI GenBank under accession numbers MZ031054–MZ031076. Appropriate accession numbers according to *Dactylogyrus* species and specific genes of rDNA regions are presented in Table 1.

## Abbreviations

18S: Small ribosomal subunit
28S: Large ribosomal subunit
5.8S: Non-coding component of the large ribosomal subunit
BI: Bayesian inference phylogenetic analysis
GTR: General time-reversible evolutionary model
ITS1: Internal transcribed spacer 1
ML: Maximum likelihood phylogenetic analysis
PCR: Polymerase chain reaction
rRNA: Ribosomal RNA

## References

[1] Brooks DR, McLennan DA (1993). Parascript: parasites and the language of evolution.

[2] Johnson KP, Adams RJ, Page RDM, Clayton DH (2003). When do parasites fail to speciate in response to host speciation?. Syst Biol.

[3] Page RDM (2003). Tangled trees: phylogeny, cospeciation and coevolution.

[4] Poulin R (2007). Evolutionary Ecology of Parasites.

[5] Ronquist F (1997). Phylogenetic approaches in coevolution and biogeography. Zool Scr.

[6] de Vienne DM, Refrégier G, López-Villavicencio M, Tellier A, Hood ME, Giraud T (2013). Cospeciation vs host-shift speciation: methods for testing, evidence from natural associations and relation to coevolution. New Phytol.

[7] Tan M, Armbruster JW (2018). Phylogenetic classification of extant genera of fishes of the order Cypriniformes (Teleostei: Ostariophysi). Zootaxa.

[8] Benovics M, Desdevises Y, Vukić J, Šanda R, Šimková A (2018). The phylogenetic relationships and species richness of host-specific *Dactylogyrus* parasites shaped by the biogeography of Balkan cyprinids. Sci Rep.

[9] Gibson DI, Timofeeva TA, Gerasev PI (1996). A catalogue of the nominal species of the monogenean genus *Dactylogyrus* Diesing, 1850 and their host genera. Syst Parasitol.

[10] Šimková A, Morand S (2008). Co-evolutionary patterns in congeneric monogeneans: a review of *Dactylogyrus* species and their cyprinid hosts. J Fish Biol.

[11] Pugachev ON, Gerasev PI, Gussev AV, Ergens R, Khotenowsky I (2009). Guide to Monogenoidea of freshwater fish of Palearctic and Amur Regions.

[12] Jarkovský J, Morand S, Šimková A (2004). Reproductive barriers between congeneric monogenean parasites (*Dactylogyrus*: Monogenea): attachment apparatus morphology or copulatory organ incompatibility?. Parasitol Res.

[13] Šimková A, Desdevises Y, Gelnar M, Morand S (2000). Co-existence of nine gill ectoparasites (Dactylogyrus: Monogenea) parasitising the roach (*Rutilus rutilus* L.): history and present ecology. Int J Parasitol..

[14] Turgut E, Shinn A, Wootten R (2006). Spatial distribution of *Dactylogyrus* (Monogenan) on the gills of the host fish. Turk J Fish Aqua Sci.

[15] Benovics M, Vukić J, Šanda R, Rahmouni I, Šimková A (2020). Disentangling the evolutionary history of peri-Mediterranean cyprinids using host-specific gill monogeneans. Int J Parasitol.

[16] Šimková A, Verneau O, Gelnar M, Morand S (2006). Specificity and specialization of congeneric monogeneans parasitizing cyprinid fish. Evolution.

[17] Fricke R, Eschmeyer WN, Van der Laan R, editors. Eschmeyer’s catalog of fishes: genera, species, references. http://researcharchive.calacademy.org/research/ichthyology/catalog/fishcatmain.asp. Accessed 15 Mar 2021.

[18] Yang L, Sado T, Vincent Hirt M, Pasco-Viel E, Arunachalm M, Li J, Wang X, Freyhof J, Saitoh K, Simons AM, Miya M, He S, Mayden RL (2015). Phylogeny and polyploidy: resolving the classification of cyprinine fishes (Teleostei: Cypriniformes). Mol Phylogenet Evol.

[19] Bănărescu PM, Bogutskaya NG (2003). The freshwater fishes of Europe, Vol. 5/II: Cyprinidae 2, Part II: *Barbus*.

[20] Levin BA, Freyhof J, Lajbner Z, Perea S, Abdoli A, Gaffaroğlu M, Özuluğ M, Rubenyan HR, Salnikov VB, Doadrio I (2012). Phylogenetic relationships of the algae scraping cyprinid genus *Capoeta* (Teleostei: Cyprinidae). Mol Phylogenet Evol.

[21] Tsigenopoulos CS, Berrebi P (2000). Molecular phylogeny of north Mediterranean freshwater barbs (genus *Barbus*: Cyprinidae) inferred from cytochrome *b* sequences: biogeographic and systematic implications. Mol Phylogenet Evol.

[22] Tsigenopoulos CS, Durand JD, Unlu E, Berrebi P (2003). Rapid radiation of the Mediterranean *Luciobarbus* species (Cyprinidae) after the Messinian salinity crisis of the Mediterranean Sea, inferred from mitochondrial phylogenetic analysis. Biol J Lin Soc.

[23] Çiçek E, Birecikligil SS, Fricke R (2015). Freshwater fishes of Turkey: a revised and updated annotated checklist. Bihar Biol.

[24] Esmaeili HR, Mehraban H, Abbasi K, Keivany Y, Coad BW (2017). Review and updated checklist of freshwater fishes of Iran: taxonomy, distribution and conservation status. Iranian J Ichthyol.

[25] Esmaeili HR, Sayyadzadeh G, Eagderi S, Abbasi K (2018). Checklist of freshwater fishes of Iran. FishTaxa.

[26] Por FD (1989). The Legacy of Tethys.

[27] Por FD, Dimentman C, Stanley DJ, Wezel FC (1985). Continuity of Messinian biota in the Mediterranean basin. Geological Evolution of the Mediterranean Basin.

[28] Durand J-D, Tsigenopoulos CS, Ünlü E, Berrebi P (2002). Phylogeny and biogeography of the family Cyprinidae in the Middle East inferred from cytochrome *b* DNA – evolutionary significance of this region. Mol Phylogenet Evol.

[29] Šimková A, Benovics M, Rahmouni I, Vukić J (2017). Host-specific *Dactylogyrus* parasites revealing new insights on the historical biogeography of Northwest African and Iberian cyprinid fish. Parasit Vector.

[30] Benovics M, Desdevises Y, Šanda R, Vukić J, Scheifler M, Doadrio I, Sousa-Santos C, Šimková A (2020). High diversity of fish ectoparasitic monogeneans (*Dactylogyrus*) in the Iberian Peninsula: a case of adaptive radiation?. Parasitology.

[31] Benovics M, Kičinjaová ML, Šimková A (2017). The phylogenetic position of the enigmatic Balkan *Aulopyge huegelii* (Teleostei: Cyprinidae) from the perspective of host-specific *Dactylogyrus* parasites (Monogenea), with a description of *Dactylogyrus omenti* n. s.. Parasit Vector..

[32] Benovics M, Desdevises Y, Šanda R, Vukić J, Šimková A (2020). Cophylogenetic relationships between *Dactylogyrus* (Monogenea) ectoparasites and endemic cyprinoids of the north-eastern European peri-Mediterranean region. J Zool Syst Evol Res.

[33] Šimková A, Morand S, Jobet E, Gelnar M, Verneau O (2004). Molecular phylogeny of congeneric monogenean parasites (*Dactylogyrus*): a case of intrahost speciation. Evolution.

[34] Šimková A, Matějusová I, Cunningham CO (2006). A molecular phylogeny of the Dactylogyridae sensu Kritsky & Boeger (1989) (Monogenea) based on the D1–D3 domains of large subunit rDNA. Parasitology.

[35] Šimková A, Pečínková M, Řehulková E, Vyskočilová M, Ondračková M (2007). *Dactylogyrus* species parasitizing European *Barbus* species: morphometric and molecular variability. Parasitology.

[36] Baguñà J, Riutort M (2004). Molecular phylogeny of the Platyhelminthes. Can J Zool.

[37] Lockyer AE, Olson PD, Littlewood DTJ (2003). Utility of complete large and small subunit rRNA genes in resolving the phylogeny of the Neodermata (Platyhelminthes): implications and a review of the cercomer theory. Biol J Linn Soc.

[38] Olson PD, Littlewood DTJ (2002). Phylogenetics of the Monogenea – evidence from a medley of molecules. Int J Parasitol.

[39] Whittington ID (2004). The Capsalidae (Monogenea: Mopisthocotylea): a review of diversity, classification and phylogeny with a note about species complexes. Folia Parasit.

[40] Roy SW, Gilbert W (2006). The evolution of spliceosomal introns: patterns, puzzles and progress. Nat Rev Genet.

[41] Scholz T, Vanhove MPM, Smit N, Jayasundera Z, Gelnar M (2018). A guide to the parasites of African freshwater fishes.

[42] Malmberg G (1957). Om forekomsten av Gyrodactylus pa svenska fiskar .Skrifter Utgivna av Sodra Sveriges Fiskeriforening. Arsskift..

[43] Blasco-Costa I, Míguez-Lozano R, Sarabeev V, Balbuena JA (2012). Molecular phylogeny of species of *Ligophorus* (Monogenea: Dactylogyridae) and their affinities within the Dactylogyridae. Parasitol Int.

[44] Šimková A, Plaisance L, Matějusová I, Morand S, Verneau O (2003). Phylogenetic relationships of the Dactylogyridae Bychowsky, 1933 (Monogenea: Dactylogyridea): The need for the systematic revision of the Ancyrocephalinae Bychowsky, 1937. Syst Parasitol.

[45] Hassouna N, Michot B, Bachellerie JP (1984). The complete nucleotide sequence of mouse 28S rRNA gene Implications for the process of size increase of the large subunit rRNA in higher eukaryotes. Nuc Acid Res..

[46] Katoh K, Misawa K, Kuma K, Miyata T (2002). MAFFT: A novel method for rapid multiple sequence alignment based on Fourier transform. Nuc Acid Res.

[47] Lanfear R, Frandsen PB, Wright AM, Senfeld T, Calcott B (2017). PartitionFinder 2: New methods for selecting partitioned models of evolution for molecular and morphological phylogenetic analyses. Mol Biol Evol.

[48] Lanfear R, Calcott B, Ho SY, Guindon S (2012). PartitionFinder: combined selection of partitioning schemes and substitution models for phylogenetic analyses. Mol Biol Evol.

[49] Stamatakis A (2006). RAxML-VI-HPC: maximum likelihood-based phylogenetic analyses with thousands of taxa and mixed models. Bioinformatics.

[50] Stamatakis A (2014). RAxML version 8: a tool for phylogenetic analyses and post-analysis of large phylogenies. Bioinformatics.

[51] Ronquist F, Teslenko M, van der Mark P, Ayres DL, Darling A, Höhna S, Larget B, Liu L, Suchard MA, Huelsenbeck JP (2012). MrBayes 3.2: efficient Bayesian phylogenetic inference and model choice across large model space. Syst Biol..

[52] Rambaut A, Drummond AJ, Xie D, Baele G, Suchard MA (2018). Posterior summarization in Bayesian phylogenetics using Tracer 1.7. Syst Biol..

[53] Maddison WP, Maddison DR. Mesquite: a modular system for evolutionary analysis. Version 3.61. 2019. http://www.mesquiteproject.org

[54] Djikanovic V, Paunovic M, Nikolic V, Simonovic P, Cakic P (2011). Parasitofauna of freshwater fishes in the Serbian open waters: a checklist of parasites of freshwater fishes in Serbian open waters. Rev Fish Biol Fisheries.

[55] Mhaisen FT, Abdul-Ameer KN (2019). Checklists of *Dactylogyrus* species (Monogenea) from Fishes of Iraq. Biol App Env Res.

[56] Moravec F (2001). Checklist of the metazoan parasites of fishes of Czech Republic and Slovak Republic (1873–2000).

[57] Pazooki J, Masoumian M (2012). Synopsis of the parasites in Iranian freshwater fishes. Iranian J Fish Sci.

[58] Abdullah YS, Abdullah SMA (2015). Some observations on fishes and their parasites of Darbandikhan lake, Kurdistan region in north Iraq. Eur Sci J.

[59] Aydoğdu N, Kubilay A (2017). Helminth fauna of Simav barbell, *Barbus niluferensis* Turan, Kottelat & Ekmekci, 2009 An endemic fish from Nilüfer river in Bursa (Turkey): new host and locality records. Bull Eur Assoc Fish Pathol.

[60] Benovics M, Francová K, Volta P, Dlapka V, Šimková A (2021). Helminth communities of endemic cyprinoids of the Apennine Peninsula, with remarks on ectoparasitic monogeneans, and description of four new *Dactylogyrus* Diesing, 1850 species. Parasitology.

[61] Gutiérrez-Galindo JF, Lacasa-Millán MI (2001). Study of the Monogenea of Cyprinidae in the Llobregat River, Northeastern Spain. II. Species composition on Barbus graellsii Steindachner, 1866. Rev Ibér Parasitol..

[62] Koyee QMK, Abdullah SMA (2019). Host specificity, community components and diversity dynamics of *Dactylogyrus* spp. (monogenean ectoparasites) parasitizing cyprinid gills. Pol J Environ Stud..

[63] Mhaisen FT, Al-Rubaie A-RL, Al-Saadi BAH (2015). Monogenean Parasites of Fishes from the Euphrates River at Al-Musaib City, Mid Iraq. Am J Biol Life Sci.

[64] Mhaisen FT, Abdullah SMA (2017). Parasites of fishes of Kurdistan region, Iraq: checklists. Biol App Env Res.

[65] Rahmouni I, Řehulková E, Pariselle A, Rkhami OB, Šimková A (2017). Four new species of *Dactylogyrus* Diesing, 1850 (Monogenea: Dactylogyridae) parasitising the gills of northern Moroccan *Luciobarbus* Heckel (Cyprinidae): morphological and molecular characterisation. Syst Parasitol.

[66] Raissy M, Ansari M (2012). Parasites of some freshwater fish from Armand River, Chaharmahal va Bakhtyari Province. Iran Iranian J Parasitol.

[67] Řehulková E, Benovics M, Šimková A (2020). Uncovering the diversity of monogeneans (Platyhelminthes) on endemic cypriniform fishes of the Balkan Peninsula: new species of *Dactylogyrus* and comments on their phylogeny and host-parasite associations in a biogeographic context. Parasite.

[68] Šimková A, Desdevises Y, Gelnar M, Morand S (2001). Morphometric correlates of host specificity in *Dactylogyrus* species (Monogenea) parasites of European Cyprinid fish. Parasitology.

[69] Shamsi S, Jalali B, Aghazadeh Meshgi M (2009). Infection with *Dactylogyrus* spp. among introduced cyprinid fishes and their geographical distribution in Iran. Iranian J Vet Res..

[70] Bănărescu P, Holcik J (1989). Zoogeography and history of the freshwater fish fauna of Europe. The freshwater fishes of Europe, vol. 1, part II.

[71] Bănărescu P (1992). Zoogeography of fresh waters, vol. 2, Distribution and dispersal of freshwater animals in North America and Eurasia.

[72] Doadrio I (1990). Phylogenetic relationships and classification of western Palearctic species of the genus *Barbus* (Osteichthyes, Cyprinidae). Aquat Living Res.

[73] El Gharbi S, Birgi E, Lambert A (1994). Monogenean Dactylogyridae parasites of Cyprinidae of the genus *Barbus* in North Africa. Syst Parasitol.

[74] Machordom A, Doadrio I (2001). Evidence of a Cenozoic Betic-Kabilian connection based on freshwater fish phylogeography (*Luciobarbus*, Cyprinidae). Mol Phylogenet Evol.

[75] Kottelat M, Freyhof J (2007). Handbook of European freshwater fishes.

[76] Řehulková R, Rahmouni I, Pariselle A, Šimková A (2021). Integrating morphological and molecular approaches for characterizing four species of *Dactylogyrus* (Monogenea: Dactylogyridae) from Moroccan cyprinids, with comments on their host specificity and phylogenetic relationships. Peer J..

[77] Kuchta R, Řehulková E, Francová K, Scholz T, Morand S, Šimková A (2020). Diversity of monogeneans and tapeworms in cypriniform fishes across two continents. Int J Parasitol.

[78] Dupont F, Lambert A (1986). Study of the parasitic communities of Monogenea Dactylogyridae from Cyprinidae in Lake Mikri Prespa (Northern Greece). Description of three new species from endemic *Barbus*: *Barbus cyclolepis prespensis* Karaman, 1924. Ann Parasitol Hum Comp.

[79] Stojanovski S, Hristovski N, Cakic P, Hristovski M (2005). Fauna of Monogenean Trematodes – parasites of some cyprinid fishes from Lake Ohrid (Macedonia). Nat Monten Podgorica.

[80] Stojanovski S, Hristovski N, Velkova-Jordanoska L, Blazekevic-Dimovska D, Atansov G (2012). Parasite fauna of Chub (*Squalius squalus* Bonaparte, 1837) from Lake Ohrid (Fyrmacedonia). Acta Zool Bulg.

[81] Machordom A, Berrebi P, Doadrio I (1990). Spanish barbel hybridization detected using enzymatic markers: *Barbus meridionalis* Risso X *Barbus haasi* Mertens (Osteichthyes, Cyprinidae). Aquat Living Res.

[82] Gante HF, Grillo O, Venora G (2011). Diversification of Circum-Mediterranean Barbels. Changing biodiversity in changing environment.

[83] Gante HF, Doadrio I, Alves MJ, Dowling TE (2015). Semi-permeable species boundaries in Iberian barbels (*Barbus* and *Luciobarbus*, Cyprinidae). BMC Evol Biol.

[84] El-Gharbi S, Renaud F, Lambert A (1992). Dactylogyrids (Platyhelminthes: Monogenea) of *Barbus* spp. (Teleostei: Cyprinidae) from Iberian Peninsula. Res Rev Parasitol..

[85] Tsigenopoulos CS, Kasapidis P, Berrebi P (2010). Phylogenetic relationships of hexaploid large-sized barbs (genus *Labeobarbus*, Cyprinidae) based on mtDNA data. Mol Phylogenet Evol.

[86] Kuljanishvili T, Epitashvili G, Freyhof J, Japoshvili B, Kalous L, Levin B, Mustafayev N, Ibrahimov S, Pipoyan S, Mumladze L (2020). Checklist of the freshwater fishes of Armenia, Azerbaijan and Georgia. J Appl Ichthyol.

[87] Mikailov TK (1974). New species of monogeneans from fishes of Azerbaijan. Parazitologia.

[88] Banarescu P, Coad BW, Winfield IJ, Nelson JE (1991). Cyprinidae of Eurasia. Cyprinid fishes, Systematics, Biology and Exploitation.

[89] Casal-Lopéz M, Doadrio I (2018). The Messinian imprint on the evolution of freshwater fishes of the genus *Luciobarbus* Heckel, 1843 (Teleostei: Cyprinidae) in the western Mediterranean. J Biogeogr.

[90] Perea S, Böhme M, Zupančič P, Freyhof J, Šanda R, Özuluğ M, Abdoli A, Doadrio I (2010). Phylogenetic relationships and biogeographical patterns in Circum-Mediterranean subfamily Leuciscinae (Teleostei, Cyprinidae) inferred from both mitochondrial and nuclear data. BMC Evol Biol.

[91] Tang QY, Getahun A, Liu HZ (2009). Multiple in–to–Africa dispersals of labeonin fishes (Teleostei: Cyprinidae) revealed by molecular phylogenetic analysis. Hydrobiologia.

[92] Yang L, Mayden RL (2010). Phylogenetic relationships, subdivision, and biogeography of the cyprinid tribe Labeonini (sensu Rainboth, 1991) (Teleostei: Cypriniformes), with comments on the implications of lips and associated structures in the labeonin classification. Mol Phylogenet Evol.

[93] Guegan JF, Lambert A, Euzet L (1988). Etude des Monogènes des Cyprinidae du genre Labeo en Afrique de l'Ouest — I Genre Dactylogyrus Diesing, 1850. Rev d'Hydrobiol Tropic..

[94] Paperna I (1973). New species of Monogenea (Vermes) from African freshwater fish. A preliminary report. Rev Zool Bot Africa.

[95] Musilová N, Řehulková E, Gelnar M (2009). Dactylogyrids (Platyhelminthes: Monogenea) from the gills of the African carp, *Labeo coubie* Rüppell (Cyprinidae), from Senegal, with descriptions of three new species of *Dactylogyrus* and the redescription of *Dactylogyrus cyclocirrus* Paperna, 1973. Zootaxa.

[96] Pravdová M, Ondračková M, Přikrylová I, Bažek R, Mahmoud Z, Gelnar M (2018). Dactylogyrids (Platyhelminthes: Monogenea) from Sudanese *Labeo* spp., with a description of *Dogielius sennarensis* n. sp. and a redescription of *Dogielius flosculus* Guégan, Lambert & Euzet, 1989. Helminthologia..

[97] Vignon M, Pariselle A, Vanhove MPM (2011). Modularity in attachment organs of African *Cichlidogyrus* (Platyhelminthes: Monogenea: Ancyrocephalidae) reflects phylogeny rather than host specificity or geographic distribution. Biol J Linn Soc.

[98] Böhme M (1995). Eine Weichschildkröte (Trionychidae) aus dem Untermiozän vom Dietrichsberg bei Vacha (Rhön). Mauritania (Altenburg).

[99] Harzhauser M, Kroh A, Mandic O, Piller WE, Göhlich U, Reuter M, Berning B (2007). Biogeographic responses to geodynamics: A key study all around the Oligo-Miocene Tethyan Seaway. Zool Anz.

[100] Otero O (2001). The oldest-known cyprinid fish of the Afro-Arabic Plate, and its paleobiogeographical implications. J Vertebr Paleontol.

[101] Van Couvering JAH (1977). Early records of freshwater fishes in Africa. Copeia.

[102] Bychowsky BE (1933). Beitrag zur Kenntnis neuer monogenetischer Fischtrematoden aus dem Kaspesee nebst einigen Bemerkungen ueber die Systematic der Monopisthodiscinea Fuhrmann, 1928. Zool Anz.

[103] Gussev AV, Ali NM, Abdul-Ameer KN, Amin SM, Molnar K (1993). New and known species of *Dactylogyrus* Diesing, 1850 (Monogenea, Dactylogyridae) from cyprinid fishes of the River Tigris. Iraq Syst Parasitol.

